# Acute central metabolic effects of otolithic vestibular stimulation in a non-human primate model of Parkinsonism: proof-of-concept neuroimaging and functional study

**DOI:** 10.3389/fneur.2026.1981761

**Published:** 2026-10-28

**Authors:** Joan Lorente-Piera, María Collantes, Ángel Ramos de Miguel, Manuel Manrique, Ángel Ramos-Macías, Sebastián Picciafuoco, Ricardo Bugallo Delgado, Irene Marcilla García, Gloria González Aseguinolaza, Purificación Ripalda-Cemboráin, José Luis Lanciego, Raquel Manrique-Huarte

**Affiliations:** 1 Department of Otorhinolaryngology, Complejo Hospitalario Universitario Insular Materno-Infantil, Las Palmas de Gran Canaria, Spain; 2 Department of Nuclear Medicine, Clínica Universidad de Navarra, Pamplona, Spain; 3 Translational Molecular Imaging Unit (UNIMTRA), Department of Nuclear Medicine, Clínica Universidad de Navarra, Pamplona, Spain; 4 IdiSNA, Instituto de Investigación Sanitaria de Navarra, Universidad de Navarra, CIMA, Pamplona, Spain; 5 Cochlear Technology Centre Belgium, Mechelen, Belgium; 6 Department of Otorhinolaryngology, Clínica Universidad de Navarra, Pamplona, Spain; 7 CNS Gene Therapy Program, Center for Applied Medical Research (Cima), University of Navarre, Pamplona, Spain; 8 Center for Biomedical Network Research on Neurodegenerative Diseases (CIBERNED-ISCIII), Madrid, Spain

**Keywords:** bilateral vestibular areflexia, bilateral vestibulopathy, neuroimaging, Parkinson‘s disease, Parkinson disease, vestibular implant

## Abstract

**Background:**

Vestibular dysfunction has increasingly been recognized in the pathophysiology of Parkinson’s disease (PD), particularly in relation to postural instability, gait impairment, and altered sensorimotor integration. However, the central metabolic effects associated with acute targeted otolithic vestibular stimulation remain poorly understood.

**Objectives:**

To explore the acute metabolic correlates associated with otolithic vestibular stimulation using ^18^F-FDG PET in a non-human primate model of PD following vestibular implantation.

**Methods:**

A unilateral otolithic vestibular implant was placed in a macaque (*Macaca fascicularis*) with MPTP-induced Parkinsonism. Parkinsonian severity and persistence of motor impairment were descriptively assessed using repeated blinded Kurlan motor scale evaluations and standardized fine motor performance tasks. Longitudinal ^18^F-FDG PET imaging was performed during four experimental stages: basal pre-MPTP condition, MPTP-induced parkinsonian state before implantation, and one- and two-month post-implantation evaluations. Post-implantation PET acquisitions were obtained under OFF and ON stimulation conditions. Voxel-wise subtraction analyses were performed to characterize relative metabolic differences across experimental stages and stimulation conditions.

**Results:**

MPTP administration induced a persistent parkinsonian phenotype characterized by marked deterioration in fine motor performance relative to baseline, together with persistently elevated Kurlan motor scores throughout follow-up. Metabolically, the MPTP-induced condition was associated with relative hypermetabolism involving especially audiovestibular cortical areas, accompanied by relative hypometabolism in frontal executive, paralimbic, and visual associative regions. Acute vestibular stimulation (ON vs. OFF) demonstrated state-dependent metabolic differences across post-implantation evaluations. During stimulation, increased metabolism predominantly involved vestibulo–thalamo–striatal, sensorimotor, and limbic–paralimbic networks. In contrast, OFF conditions showed relatively greater involvement in visual associative cortices and cerebellar vermis.

**Conclusion:**

Acute otolithic vestibular stimulation was associated with metabolic modulation of vestibulo–thalamo–striatal and sensorimotor-related networks in a parkinsonian non-human primate model. These findings provide proof-of-concept evidence supporting the capacity of vestibular neurostimulation to engage distributed central motor integration circuits.

## 1 Introduction

Parkinson’s disease (PD) is a progressive neurodegenerative disorder, characterized by a motor syndrome comprising bradykinesia, rigidity, tremor, and postural instability. Although degeneration of the nigrostriatal dopaminergic pathway is central to its pathophysiology, PD is increasingly recognized as a multisystem disorder in which non-dopaminergic networks contribute substantially to both motor and non-motor manifestations (1, 2). Postural instability and gait disturbances—among the most disabling and treatment-resistant manifestations—suggest the involvement of sensory–motor systems beyond the basal ganglia (3).

The vestibular system plays a critical role in postural control, gait, and visuomotor stabilization (3). It comprises the semicircular canals, which encode angular acceleration, and the otolith organs, which encode linear acceleration and gravity. Beyond its classical role in spatial orientation, the otolith system—particularly the saccular pathway—has been proposed to contribute to motor control by influencing central sensorimotor integration (4). Accordingly, vestibular dysfunction may contribute to postural instability and gait impairment in PD. (5) When vestibular input is impaired, postural control can be partially maintained through sensory reweighting, with increased reliance on visual and somatosensory information. Visual cues may provide an alternative reference for spatial orientation and Earth vertical, thereby contributing to stabilization of the head and trunk when vestibular information is unreliable. However, excessive reliance on visual information may also result in visual dependence, particularly when visual and vestibular cues are incongruent (6, 7).

Neurovestibular abnormalities have been described in PD and atypical Parkinsonism, and vestibular impairment has been associated with falls and postural instability (2, 3, 8). From an anatomical perspective, vestibular nuclei project to thalamic relay nuclei, including the parafascicular nucleus, which in turn connects with the dorsolateral putamen, a key component of basal ganglia motor-related circuitry. These vestibulo–thalamo–striatal pathways provide a plausible structural substrate through which vestibular inputs may modulate motor networks (9, 10).

Further support for vestibular–motor interactions derives from observations in patients with cerebellar disorders or bilateral vestibular dysfunction, who frequently exhibit motor deficits resembling those seen in PD, including gait ataxia, impaired coordination or even oscillopsia and risk of falls (11, 12). In parallel, direct electrical otolith-targeted vestibular stimulation delivered through an implantable vestibular device has been shown to induce measurable central effects, reinforcing the concept that vestibular inputs can modulate distributed brain networks involved in sensorimotor processing (13).

Within this conceptual framework, the BionicVEST project was developed to restore vestibular function through direct electrical stimulation of the otolith organs using an implantable vestibular device. Initial human studies demonstrated the feasibility and safety of otolith stimulation and its capacity to restore vestibular function (14, 15). Notably, one of the subjects implanted within this program presented not only with bilateral vestibulopathy but also with comorbid PD, and showed improvements extending beyond the audiovestibular domain, including functional performance, quality of life, and supratentorial metabolic patterns following implantation, differing from those observed in peers with other etiologies (13, 16). These observations provided a key rationale for the present study and underscored the need for objective biomarkers capable of characterizing central effects of vestibular stimulation.

Non-animal models and lower phylogenetic species are insufficient to address these combined objectives, as they do not allow replication of the human surgical approach, vestibular electrode implantation, or simultaneous evaluation of central metabolic effects and auditory function. In contrast, non-human primates provide a highly relevant translational platform due to their close similarity to humans in temporal bone anatomy, inner ear histology, and auditory physiology (17, 18). Moreover, 1-methyl-4-phenyl-1,2,3,6-tetrahydropyridine (MPTP)-induced Parkinsonism in macaques reproduces several key pathological and functional features of human PD and remains one of the most widely used translational models in experimental neuroscience (19, 20). We hypothesized that acute otolithic vestibular stimulation could be associated with condition-dependent changes in cerebral glucose metabolism within predefined sensorimotor and vestibulo-related networks in a parkinsonian non-human primate model, detectable by functional neuroimaging.

The primary objective of this proof-of-concept study was to explore and descriptively characterize the central metabolic patterns associated with acute otolithic vestibular stimulation using 2-[^18^F]-fluoro-2-deoxy-D-glucose ([^18^F]-FDG) PET (FDG-PET) in a parkinsonian non-human primate model. Additionally, we aimed to preliminarily assess the feasibility of combining vestibular neurostimulation, functional neuroimaging, and auditory preservation analysis within a translational experimental framework.

## 2 Materials and methods

In the present study, we conducted a proof-of-concept experimental animal investigation following vestibular implantation in a non-human primate model of PD (*Macaca fascicularis,* Mf) chronically treated with the dopaminergic neurotoxin MPTP.

### 2.1 Experimental animal

The study was conducted in a young adult male cynomolgus monkey (*M. fascicularis*), weighing between 4 and 5 kg. The animal was housed and cared for at the animal facilities of the University of Navarra under standardized environmental conditions, including controlled humidity (50%), air exchange (16 L/min), and a 12-h light/dark cycle (lights on from 8:00 a.m. to 8:00 p.m.). The animal received a diet consisting of fresh fruit and commercial primate pellets and had ad libitum access to water. The choice of this macaque species was based on its close evolutionary relationship to humans, making it the gold-standard model and a well-established translational model that bridges fundamental experimental research and clinically relevant applications. As previously demonstrated, the surgical approach employed closely mirrors that used in human procedures (21), which is essential for achieving the study objectives and for ensuring the translational relevance of the findings to clinical practice.

### 2.2 Induction of Parkinsonism

After the quarantine period, the animal was trained to perform fine motor tasks with each hand separately. Parkinsonism was induced by weekly intravenous injections of MPTP 0.3 mg/kg under ketamine anesthesia until, at least, a moderate and persistent parkinsonian phenotype was achieved. A 4-week washout period followed to ensure stabilization. Severity and persistence were assessed weekly and defined as a score of at least 20 points out of a maximum of 29 on the Kurlan motor scale (22) by two experienced observers under blinded conditions to minimize observer-related bias. For analysis, the mean score across the observation period was used to characterize the persistence of parkinsonian impairment.

### 2.3 Motor evaluation of the response to levodopa and fine motor performance

Motor performance was evaluated at four time points: baseline (Basal), pre-operative (parkinsonian state), 1 month post-operative (implant OFF), and 2 months post-operative (implant OFF). At each time point, the animal received oral levodopa/carbidopa (10 mg/kg) after a 12-h fast, and motor assessments were performed every 10 min for 2 h using a primate-specific Parkinsonism rating scale (23, 24). All sessions were video-recorded for blinded offline evaluation. Placebo sessions (orange juice only) were conducted separately. For descriptive analysis, the mean motor score across the 2-h observation window was used. These assessments served to confirm a persistent, levodopa-responsive parkinsonian phenotype prior to neuroimaging.

Fine motor performance was assessed at the same time points using a standardized object retrieval task (23). The animal was required to retrieve four peanuts with each hand, across five consecutive trials per session. The mean retrieval time (in seconds) for each hand was calculated across trials. The same protocol was repeated after placebo administration. Trials not completed were excluded from analysis. As with the global Parkinsonism rating scale, this task was included to further characterize the severity and dopaminergic sensitivity of motor impairment following MPTP exposure and was not intended to assess changes in motor performance related to vestibular stimulation.

### 2.4 Anesthesia procedure

According to the protocol established for this species, the animal was anesthetized under a multimodal analgesia regimen and continuously monitored throughout the procedure (25). General immobilization was induced with an intramuscular combination of atropine sulfate (0.05 mg/kg), midazolam (0.5 mg/kg), and ketamine (5 mg/kg). Prior to the initial incision, an intravenous bolus of fentanyl (10 μg) was administered.

General anesthesia was maintained with a mixture of nitrous oxide and oxygen (50%), sevoflurane (2–3%), and continuous opioid analgesia using either fentanyl infusion (2–4 μg/kg/h) or remifentanil (Ultiva®, 0.5–0.9 μg/kg/min). Corticosteroids were not administered systematically during the postoperative or follow-up periods, as their use could interfere with the natural inflammatory response.

### 2.5 Surgical procedure

The animal electrode array (HL03) was designed by Cochlear Ltd., Sydney, Australia, and consists of a half-banded, three-contact intravestibular array: 2.25 mm in length, 0.15 mm in width at the tip, and 0.64 mm in the widest part (base). The distance between the tip and the apical electrode (E3) is 0.35 mm. Each electrode had a length of 0.25 mm. The distance between the tip of the implant and the basal end of the first electrode (E1) was 1.6 mm. All parameters shown in the Figure 1 are in mm. The intravestibular array tapers to a wider extravestibular helix lead wire insulated with silicone. The intravestibular and two reference electrodes were connected to a transcutaneous 6-pin connector.

**Figure 1:**
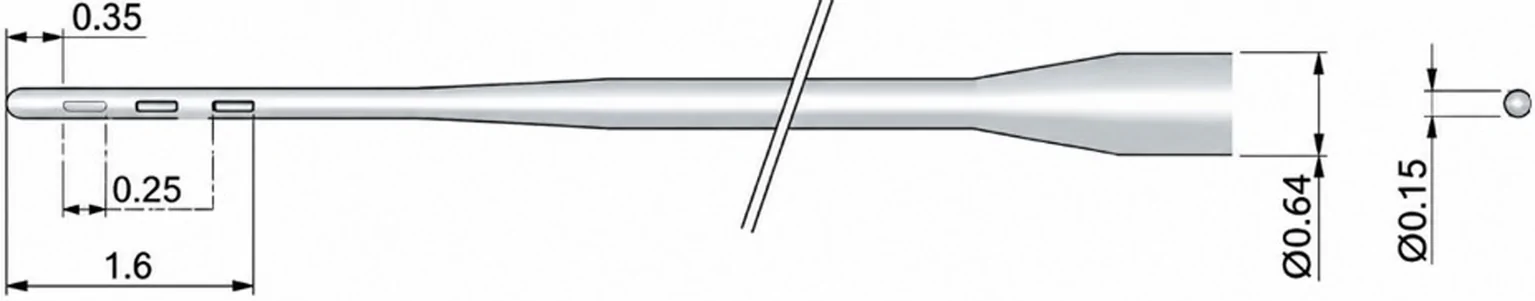
Schematic representation of the vestibular electrode array used for otolithic stimulation. Ø, diameter.

Implantation was performed unilaterally by a senior surgeon. After removal of the posterior crus of the stapes, vestibular access was achieved by creating a 0.5-mm stapedotomy using a CO₂ laser (AcuPulse DUO, Lumenis Ltd., Israel) and directed medially and inferiorly toward the anterior crus of the stapes. The vestibular electrode was inserted to a maximum depth ranging between 2.2 and 2.3 mm, corresponding to the insertion of two contacts of the vestibular array. This anatomical trajectory was designed to preferentially target the inferior vestibular nerve and saccular region. However, anatomical proximity does not demonstrate exclusive activation of otolithic afferents, and some degree of current spread with co-activation of neighboring vestibular structures cannot be excluded.

Stimulation consisted of charge-balanced biphasic cathodic first pulse trains with a pulse phase width of 25 μs and a stimulation rate of 900 pulses per second. Monopolar stimulation was delivered using the ball and plate electrodes as extracochlear references (MP1 configuration). Stimulation delivery differed according to the target structure: stimulation was amplitude-modulated within the dynamic range defined between threshold (T) and comfort (C) levels, whereas otolithic stimulation was delivered at a fixed current amplitude (T = C).

### 2.6 ^18^F-FDG PETacquisition and experimental conditions

The animal underwent ^18^F-FDG PET imaging to assess longitudinal cerebral metabolic changes associated with MPTP-induced Parkinsonism, vestibular implantation, and acute otolith-targeted vestibular stimulation. PET studies were acquired at four predefined experimental stages: (1) Basal pre-MPTP condition, (2) MPTP-induced parkinsonian condition before implantation, (3) 1 month post-implantation, and (4) 2 months post-implantation.

During both pre-implantation stages, imaging was performed under resting conditions in the absence of vestibular stimulation. At each post-implantation stage, two PET acquisitions were obtained under different stimulation conditions (Figure 2). In the OFF condition, the vestibular implant was deactivated at least 2 h before imaging to minimize residual stimulation effects, according to our previous vestibular implantation protocol (13, 16). In the ON condition, the right-sided otolith-targeted vestibular electrode array remained active throughout the FDG uptake period, using identical stimulation parameters across sessions. No externally imposed visual, auditory, motor, or task-related stimulation was provided during any acquisition.

**Figure 2:**
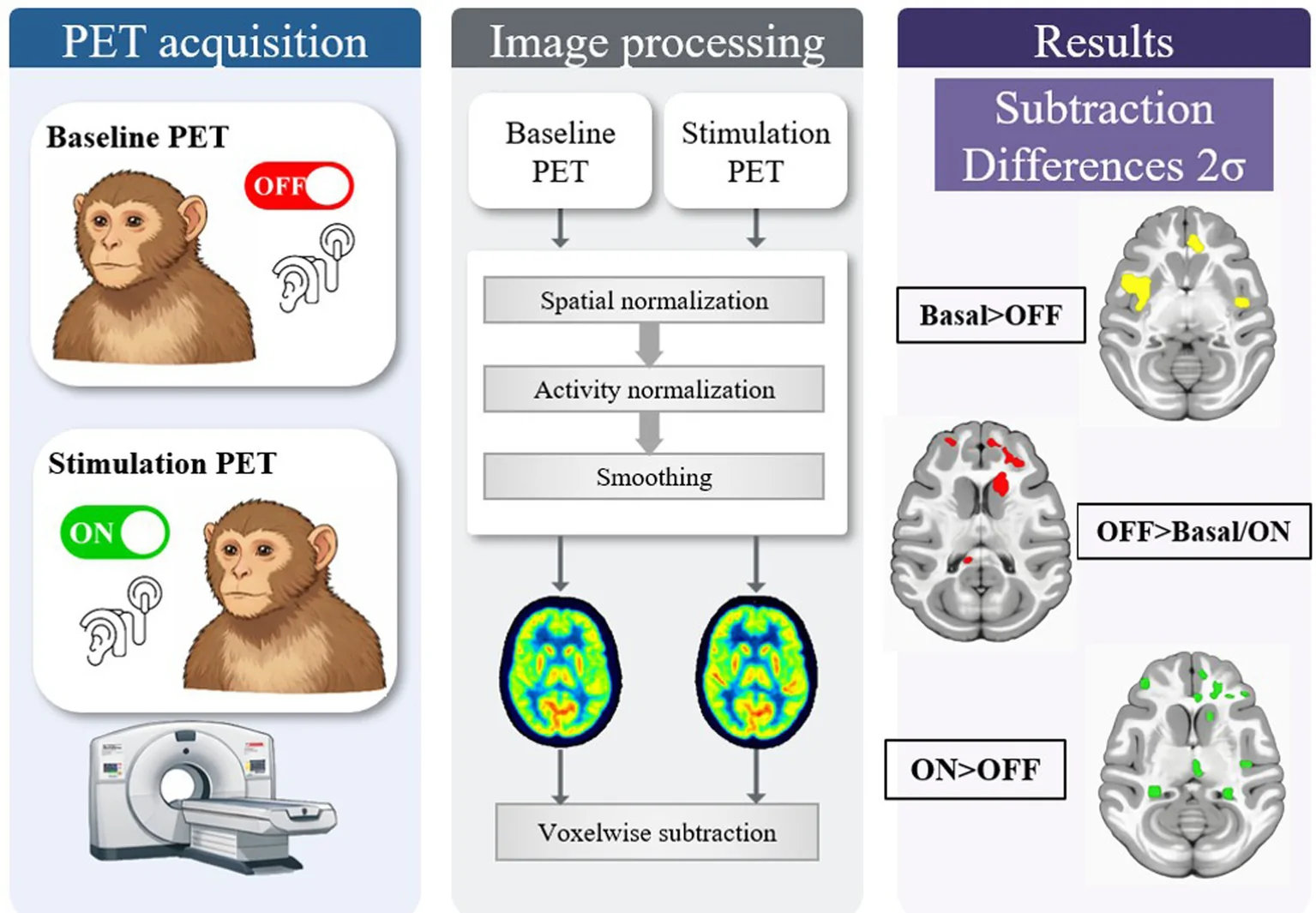
Summary of the experimental design and FDG PET comparison strategy.

This experimental design allowed two independent types of metabolic comparison. Disease-related changes were assessed by comparing the Basal pre-MPTP and MPTP-induced pre-implantation conditions, whereas the acute metabolic effects associated with vestibular stimulation were explored through OFF versus ON comparisons at one and 2 months post-implantation. No direct voxel-wise comparisons were performed between the Basal condition and post-implantation ON or OFF conditions. Accordingly, the labels in Figure 2 indicate metabolic directionality only within each independent comparison.

PET acquisitions followed a standardized protocol adapted from that previously described for longitudinal metabolic imaging in MPTP-treated non-human primates (26). The animal was anesthetized before transfer to the PET facility with intramuscular ketamine (10 mg/kg) and midazolam (0.5 mg/kg). The time of anesthetic administration was recorded, and half of the initial anesthetic dose was subsequently administered every 30 min until completion of the procedure.

^18^F-FDG was administered intravenously, with an injected activity of 79.7 ± 4.7 MBq across the seven acquisitions (range, 74.0–85.1 MBq). Pre-injection blood glucose values, available for six of the seven acquisitions, were 68.3 ± 3.7 mg/dL (range, 63–74 mg/dL); glucose was not recorded during the initial Basal acquisition because a glucometer was unavailable.

During the 40-min FDG uptake period, the animal remained anesthetized and at rest in the prone position on the scanner bed and was covered with a blanket to minimize environmental and physiological variability. Cardiorespiratory rate and body temperature were manually assessed by trained personnel before transfer from the animal facility and upon arrival at the PET facility. No continuous instrumental physiological monitoring was performed inside the PET scanner; however, the animal’s clinical status and depth of anesthesia were periodically assessed throughout the procedure.

Following the 40-min uptake period, a 20-min static emission scan was acquired with the animal maintained in the prone position using a Mosaic PET scanner (Philips). The anesthesia protocol, uptake duration, resting conditions, animal positioning, scanner, and acquisition procedure were maintained consistently across longitudinal examinations and, particularly, between paired post-implantation ON and OFF acquisitions.

### 2.7 ^18^F-FDG PET image processing and exploratory voxel-wise analysis

PET images were reconstructed and processed using PMOD software (version 4.2). Voxel values were converted to standardized uptake values (SUV) by normalization to the injected radiotracer activity and the animal’s body weight. All images were spatially normalized to the NIH Macaque Template (NMT, version 2.0), a high-resolution standardized anatomical reference for the macaque brain, to ensure anatomical correspondence across conditions and time points (27).

Voxel-wise analyses were performed as exploratory within-subject comparisons to characterize the spatial distribution of relative FDG uptake differences between experimental conditions. For each comparison, voxel-by-voxel SUV subtraction maps were generated using a custom-written MATLAB-based program. The distribution of inter-condition SUV differences was calculated across voxels within the whole-brain mask, and its standard deviation (SD) was used to define a descriptive visualization threshold. Voxels showing an absolute SUV difference greater than two SDs of the corresponding whole-brain inter-condition difference distribution were retained to visualize the largest condition-dependent metabolic differences.

The 2-SD criterion was used exclusively as an exploratory descriptive threshold and was not considered a test of voxel-wise statistical significance. Accordingly, no voxel-wise *p*-values were calculated, no correction for multiple comparisons was applied, and spatial dependence among neighboring PET voxels was not explicitly modeled. The resulting maps were therefore interpreted as hypothesis-generating representations of within-subject metabolic differences rather than as statistically significant regional effects. For visualization, relative metabolic differences were color-coded according to the direction of each independent comparison:

- *Yellow*: Higher metabolism in the baseline situation compared to parkinsonian pre-implant condition (Basal>MPTP-induced)
- *Red*: Higher metabolism in the pre-operative condition (MPTP-induced>Basal) as well as in the post-operative condition (OFF > ON).
- *Green*: Higher metabolism in the post-operative condition during vestibular stimulation (ON > OFF).

As illustrated in Figure 3, the resulting spatial patterns were anatomically contextualized using a predefined network-based framework comprising cortical and subcortical regions involved in vestibular processing, multisensory integration, motor control, and limbic–paralimbic functions, based on previous functional neuroimaging studies and our prior work with vestibular implantation models (13). This framework was used exclusively to guide anatomical interpretation and did not constitute region-of-interest or network-level statistical testing.

**Figure 3:**
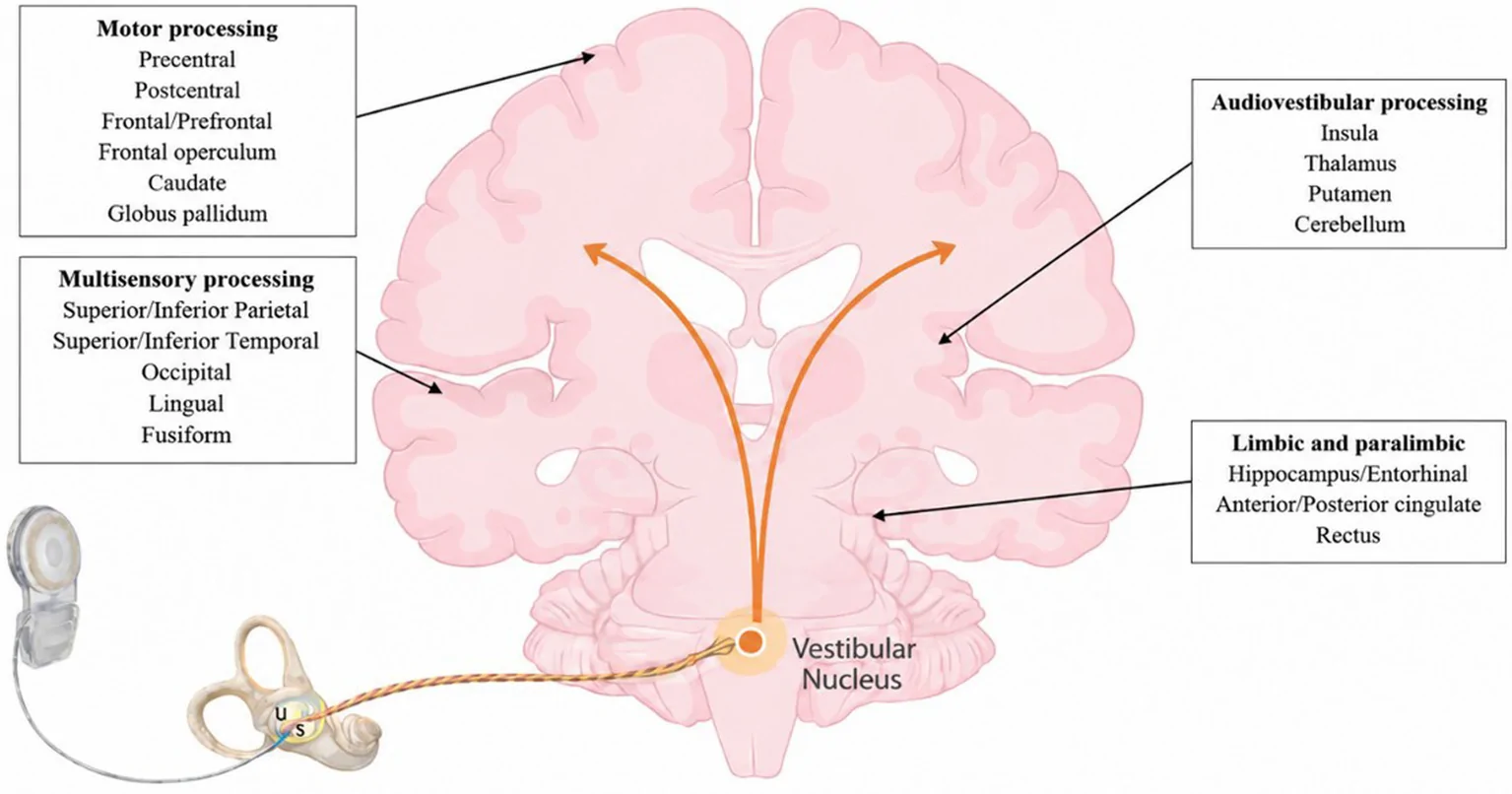
Schematic representation of the functional networks showing condition-dependent metabolic modulation following otolith vestibular stimulation. Brain regions were grouped at the network level into motor processing, multisensory processing, audiovestibular processing, and limbic/paralimbic networks. Arrows illustrate the propagation of vestibular input from the vestibular nuclei toward distributed cortical and subcortical networks. S, Saccule; U, Utricle.

### 2.8 Euthanasia and tissue processing

At the end of the study, the animal was euthanized by intracardiac perfusion. Perfusion began with Ringer’s saline solution, followed by 3,000 mL of a buffered fixative solution containing 4% paraformaldehyde and 0.1% glutaraldehyde. This was followed by perfusion with a cryoprotective solution composed of 10% glycerin and 1% dimethyl sulfoxide (DMSO) in 0.1 M phosphate buffer at neutral pH. After completion of perfusion, the brains were removed from the skulls and stored for 48 h in a second cryoprotective solution containing 20% glycerin and 2% DMSO in 0.1 M phosphate buffer (pH 7.4).

### 2.9 Statistical analysis

Given the single-subject design, behavioral analyses were descriptive and no inferential statistical testing was performed. The animal constituted the biological experimental unit (n = 1), while repeated trials obtained within each assessment session were considered within-subject observations rather than independent biological replicates. Fine motor performance was summarized across trials at each experimental stage (Basal, Pre-Operative, 1-month Post-Operative, and 2-month Post-Operative) to characterize the induction and longitudinal persistence of the parkinsonian motor phenotype. These behavioral assessments were not designed to evaluate the therapeutic efficacy of vestibular stimulation.

## 3 Results

The surgical procedure was successfully performed on the right ear of Mf. No complications during surgical or anesthetic procedures were observed. A cumulative MPTP dose of 5.7 mg/kg was finally reached.

### 3.1 Motor performance across experimental stages

Consistently, preoperative assessments by both blinded observers yielded a mean Kurlan motor score of 22.67 ± 0.82 points during the MPTP pre-implantation stage, compared with 20.75 ± 1.26 points at 1 month post-implantation and 19.75 ± 0.50 points at 2 months post-implantation, further supporting the relative longitudinal stability of parkinsonian severity from a clinical perspective. Importantly, all postoperative motor evaluations were performed with the vestibular implant deactivated (OFF condition), thereby excluding acute stimulation-related effects during behavioral assessment.

As shown in Figure 4, fine motor performance deteriorated markedly after MPTP administration compared with the basal pre-MPTP condition, confirming the successful induction of a parkinsonian motor phenotype. Retrieval times remained consistently elevated throughout all subsequent evaluations, both before and after implantation, supporting the persistence of motor impairment across the experimental timeline. Repeated assessments performed at each stage showed persistent motor impairment, with clear separation from the basal condition.

**Figure 4:**
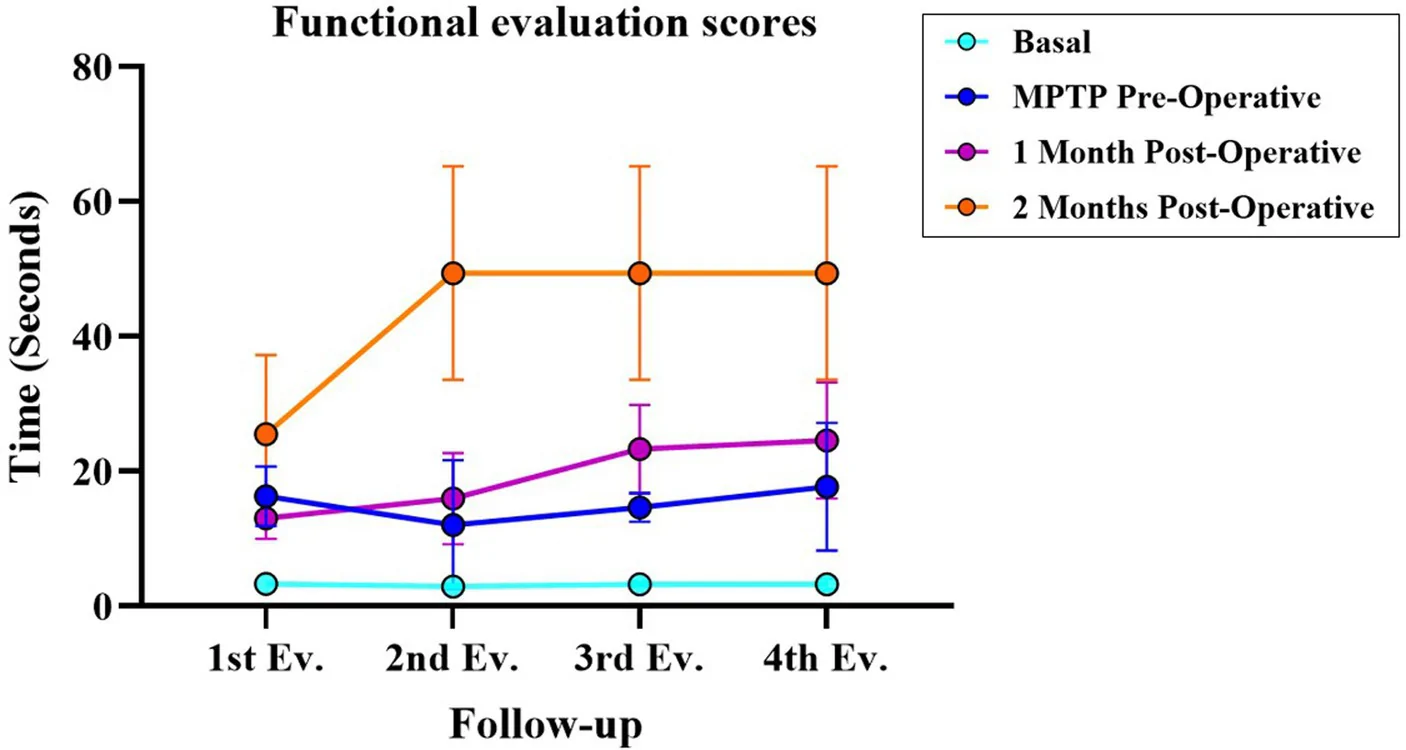
Fine motor performance across experimental conditions and follow-up evaluations. Ev: Evaluation.

### 3.2 PET results

#### 3.2.1 Pre-implantation: basal versus MPTP condition

Comparison between the basal pre-implantation state and the MPTP-induced parkinsonian condition revealed regionally specific metabolic differences, reflecting the impact of dopaminergic lesioning on distributed cortical and subcortical networks. Corresponding metabolic patterns are summarized in Figure 5 and Table 1.

**Figure 5:**
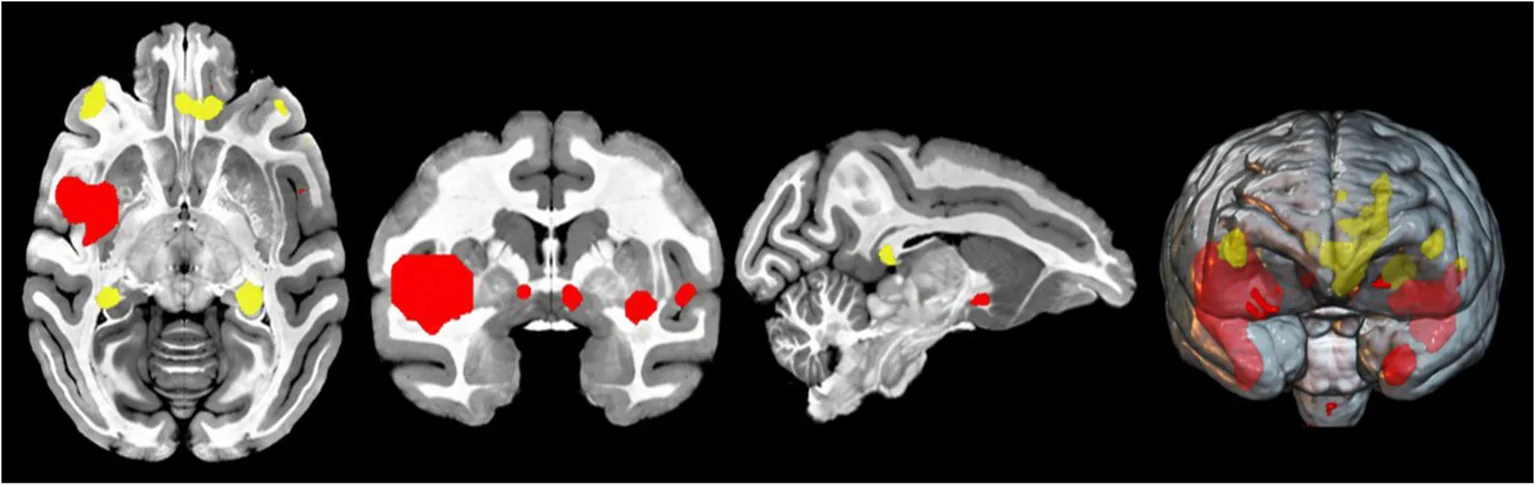
Voxel-wise ^18^F-FDG PET comparison between the basal pre-implantation condition and the MPTP-induced parkinsonian state in Mf1, displayed in axial, coronal, sagittal, and 3D-rendered views normalized to the NMT space. Voxels showing higher metabolism in the basal condition relative to MPTP (Basal > MPTP) are displayed in yellow, whereas voxels with higher metabolism during the MPTP-induced Parkinsonism (MPTP > Basal) are displayed in red (threshold > 2 SD).

**Table 1:** Summary matrix of condition-dependent ^18^F-FDG PET metabolic changes across functional brain networks.

Anatomical regions		Basal vs MPTP		OFF vs ON 1 Month		OFF vs ON 2 Months	
		IPSIL	CONTRA	IPSIL	CONTRA	IPSIL	CONTRA
Audio-vestibular processing	Insula						
	Thalamus						
	Putamen						
	Cerebellum						
Motor- executive processing	Precentral						
	Postcentral						
	Frontal-Prefrontal						
	Frontal Operculum						
	Caudate						
	Globus pallidum						
Multisensory processing	Parietal						
	Temporal						
	Occipital						
	Lingual						
	Fusiform						
Limbic emotional processing	Hippocampus						
	Cingulate						
	Rectus						

IPSIL, Ipsilateral; CONTRA, Contralateral. Yellow: Higher metabolism in Basel pre-MPTP conditional (Basal > MPTP-induced). Red: Higher metabolism in the reference condition (MPTP-induced > ON depending on the comparison). Green: Higher metabolism during active vestibular stimulation (ON > OFF). White: No relevant metabolic difference detected above the predefined threshold.

Relative hypermetabolism in the basal condition (Basal > MPTP) was predominantly observed in bilateral frontal and prefrontal cortex, including the gyrus rectus and frontal operculum, as well as in bilateral occipital and secondary visual cortex involving the lingual and fusiform gyri. This pattern was characterized by relatively higher metabolic engagement of frontal executive, paralimbic, and visual associative networks prior to MPTP-induced dopaminergic disruption.

In contrast, the MPTP condition (MPTP > Basal) was characterized by increased metabolism in sensorimotor and audiovestibular-related regions, including the precentral gyrus, insula, temporal cortex, putamen, and thalamus. This distribution aligns with disease-related reorganization of striato–thalamo–cortical circuits and altered sensory–motor integration associated with parkinsonian pathophysiology.

#### 3.2.2 Post-implantation: 1 month OFF vs. ON

As shown in Figure 6 and Table 1, at one-month post-implantation, acute activation of the vestibular implant (ON vs. OFF) was associated with focal metabolic modulation across distributed cortical and subcortical networks.

**Figure 6:**
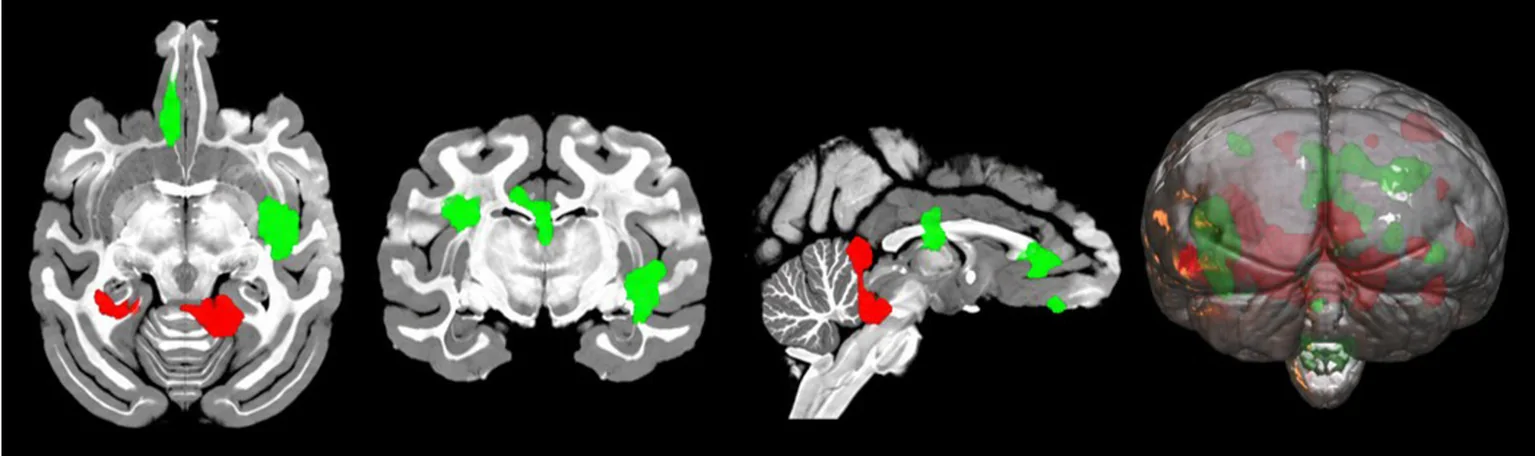
Voxel-wise ^18^F-FDG PET comparison at 1 month post-implantation contrasting vestibular stimulation ON and OFF conditions in Mf1, shown in axial, coronal, sagittal, and 3D-rendered views. Voxels with higher metabolism during active stimulation (ON > OFF) are shown in green, while voxels with higher metabolism during the OFF condition (OFF > ON) are shown in red (threshold > 2 SD).

During the OFF condition (OFF > ON), relative hypermetabolism was predominantly observed in associative and integrative cortical regions, including bilateral lingual and fusiform gyri and broader occipital cortex involvement. Additional subcortical and infratentorial engagement was detected, particularly in bilateral cerebellar vermis. This metabolic profile suggests a relative dominance of higher-order visuospatial and associative processing, accompanied by cerebellar involvement, when vestibular stimulation is absent.

Conversely, during the ON condition (ON > OFF), increased metabolism was primarily detected within motor, audiovestibular-related and limbic-paralimbic circuits. These included the bilateral frontal and prefrontal cortices, frontal operculum and insula, the bilateral precentral and postcentral gyri, bilateral anterior cingulate cortex, bilateral putamen and pallidum, the caudate nucleus and thalamus, as well as the cingulate and bilateral rectus gyri. These findings suggest preferential engagement of vestibulo–thalamo–striatal and cerebello–motor networks during active otolithic stimulation, with relative redistribution of metabolic activity toward subcortical sensorimotor integration networks during active stimulation.

#### 3.2.3 Post-implantation: 2 months OFF vs. ON

At 2 months post-implantation, OFF vs. ON comparisons revealed more circumscribed metabolic patterns, as summarized in Figure 7 and Table 1.

**Figure 7:**
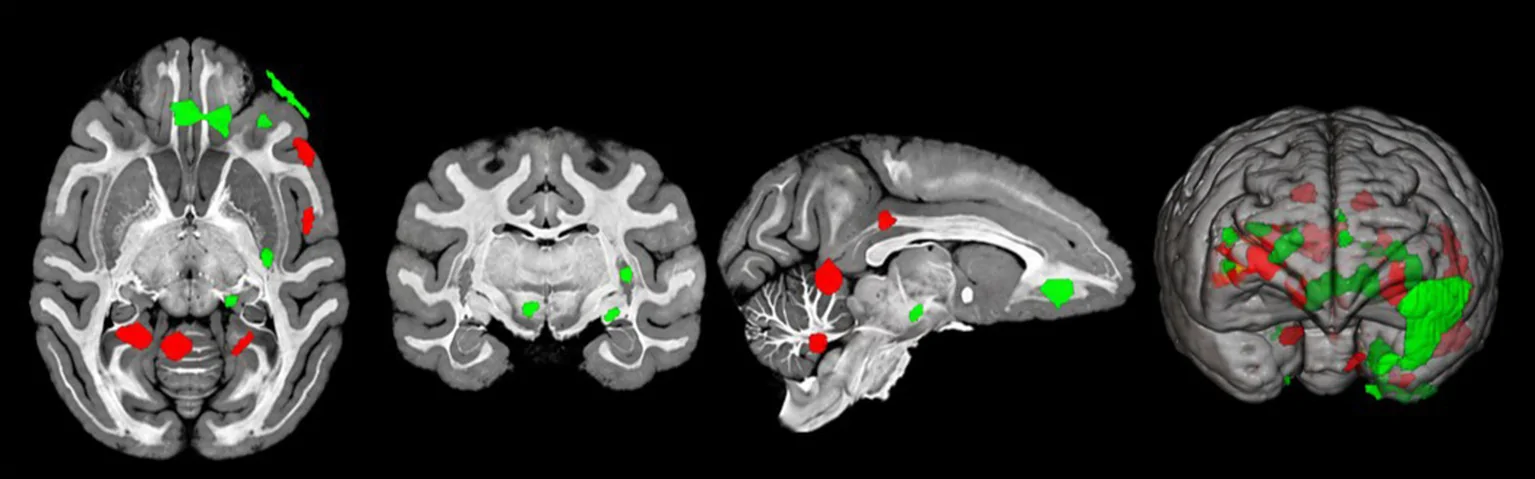
Voxel-wise ^18^F-FDG PET comparison at 2 months post-implantation contrasting vestibular stimulation ON and OFF conditions in Mf1, shown in axial, coronal, sagittal, and 3D-rendered views. Voxels with higher metabolism during active stimulation (ON > OFF) are shown in green, while voxels with higher metabolism during the OFF condition (OFF > ON) are shown in red (threshold > 2 SD).

Similar to the first-month findings, the OFF condition (OFF > ON) continued to show relative hypermetabolism in frontal operculum, bilateral occipital and secondary visual cortices (lingual and fusiform gyri), cerebellar vermis, and exploratory involvement of insula and hippocampal and entorhinal regions. This pattern was characterized by continued involvement of visual and limbic–paralimbic regions during the absence of vestibular stimulation.

During the ON condition (ON > OFF), increased metabolism was observed mainly in bilateral prefrontal cortices, bilateral anterior cingulate, rectus gyri, pre and postcentral cortex, putamen, parieto-temporal cortex, and bilateral thalamus. These findings indicate sustained modulation of fronto-striatal, thalamo-cortical, and sensorimotor regions during ongoing vestibular stimulation.

Overall, ON–OFF comparisons demonstrated state-dependent metabolic differences preferentially involving motor, striatal, and audiovestibular-related regions during vestibular stimulation, together with relative involvement of frontal associative and visual networks during the OFF condition.

## 4 Discussion

This study provides a translational evaluation of otolithic vestibular stimulation in a non-human primate model of PD, integrating functional neuroimaging within a controlled experimental framework. The results should be interpreted as a single-subject proof-of-concept showing condition-dependent metabolic differences across vestibulo–thalamo–striatal and sensorimotor-related regions during acute otolith-targeted vestibular stimulation. In contrast to prior human observations under chronic stimulation paradigms (13), the present data capture an acute stimulation model, thereby offering insight into the early network-level effects that may precede long-term neuroplastic adaptation.

From a pathophysiological perspective, vestibular dysfunction has increasingly been recognized as a relevant contributor to both motor and non-motor manifestations of PD, including postural instability, gait impairment, and altered sensory integration (7). Experimental and clinical studies have demonstrated that vestibular inputs project through thalamic relay nuclei toward basal ganglia structures, providing an anatomical substrate through which vestibular signals may influence motor control circuits (8, 10). In this context, previous neuromodulation approaches such as deep brain stimulation (DBS) (28–30) and galvanic vestibular stimulation (31, 32) have shown that modulation of sensory or subcortical input can influence gait and postural control, although the underlying mechanisms remain incompletely understood and often lack network-level specificity. Moreover, several studies have incorporated ^18^F-FDG PET imaging to characterize metabolic changes associated with DBS and to explore imaging-based predictors of therapeutic response (33, 34). The present findings extend this framework by suggesting that targeted otolithic stimulation may also engage vestibulo–thalamo–striatal and sensorimotor networks within a controlled and anatomically targeted framework.

Comparison between the basal pre-lesion condition and the MPTP-induced parkinsonian state revealed a metabolic redistribution consistent with established PD physiology (35–38). Following MPTP administration, increased glucose metabolism was observed in the putamen, thalamus, insula, and sensorimotor cortices, accompanied by relative reductions in prefrontal and posterior associative regions. Similar patterns have been described in idiopathic PD and are commonly interpreted as reflecting compensatory metabolic load within inefficient striato–thalamo–cortical circuits rather than preserved function (39, 40). Importantly, both the putamen and thalamus are key nodes for multisensory integration, including vestibular afference. Anatomical studies have demonstrated disynaptic projections from vestibular nuclei to thalamic relay nuclei and subsequently to the dorsolateral putamen, providing a structural substrate through which vestibular input can influence basal ganglia output (41).

Within this pathophysiological framework, a key observation of the present study is the different metabolic distribution observed between the ON and OFF conditions under acute otolith-targeted vestibular stimulation. During active stimulation, relatively greater FDG uptake involved the thalamus, basal ganglia, sensorimotor cortex, and anterior cingulate, indicating metabolic engagement of regions implicated in vestibular and sensorimotor processing (42–44). These regions participate in the integration of vestibular and gravito-inertial information with motor processing; however, the present imaging paradigm cannot determine whether their relative recruitment represents compensatory processing, increased computational demand, or another form of network response.

Conversely, the OFF condition showed relatively greater metabolic activity in posterior associative and visual regions, including the occipital cortex and lingual and fusiform gyri. Visual and multisensory networks have previously been implicated in both bilateral vestibular loss and PD-related postural dysfunction (45, 46). Nevertheless, the present data cannot establish whether the relative differences between ON and OFF conditions reflect compensatory visual reliance or a beneficial redistribution of processing during vestibular stimulation.

Thus, the ON–OFF differences should primarily be interpreted as evidence of condition-dependent metabolic engagement of distinct distributed networks. Whether these patterns reflect bottom-up vestibular integration, compensatory reorganization, maladaptive recruitment, or functionally relevant network modulation cannot be determined from FDG-PET alone and will require complementary functional and electrophysiological investigation.

Within this framework, the functional motor data presented in Figure 4 should be interpreted with caution. These analyses were not designed to assess therapeutic efficacy of vestibular implantation. Instead, their purpose was to confirm the successful induction and longitudinal persistence of the parkinsonian phenotype following MPTP administration. The persistence of prolonged retrieval times and persistently elevated Kurlan scores pre- and post-operative conditions, despite modest longitudinal variation, demonstrates that the underlying motor deficit remained intact throughout the study period. This is a necessary condition for interpreting the PET findings, as it ensures that observed metabolic changes are not confounded by spontaneous recovery or fluctuations in disease severity. Accordingly, the absence of functional improvement is not a negative result but rather a methodological requirement that reinforces the internal validity of the model.

The absence of a direct functional correlate between metabolic modulation and motor performance should therefore be understood in light of the study objectives. This work was not intended to evaluate clinical benefit, but rather to isolate and characterize the central effects associated with an otolith-targeted vestibular stimulation paradigm. The bilateral cortical metabolic changes observed during stimulation are compatible with the widespread vestibulo–thalamo–cortical projections described anatomically and may provide a framework for interpreting the broader supratentorial effects reported in previous human vestibular implantation studies (47, 48).

Several limitations must be acknowledged. The major limitation of this study is that longitudinal FDG-PET assessment was feasible in a single non-human primate due to the logistical and ethical constraints inherent to primate research, and the findings should therefore be interpreted as hypothesis-generating rather than inferential. Secondly, the PET analysis relies on voxel-wise subtraction without formal correction for multiple comparisons, and the thresholding strategy is inherently exploratory Furthermore, although the electrode trajectory was anatomically directed toward the inferior vestibular nerve and saccular region, the present study cannot demonstrate exclusive activation of otolithic afferents. Current spread and unintended co-activation of neighboring vestibular structures cannot be excluded. Despite these limitations, the study provides preliminary *in vivo* evidence that otolith-targeted vestibular stimulation can be associated with condition-dependent metabolic changes across vestibulo–thalamo–striatal and sensorimotor-related regions. These hypothesis-generating observations provide a rationale for future studies incorporating biological replication, chronic stimulation paradigms, and quantitative network analyses.

## 5 Conclusion

This single-subject proof-of-concept study shows that acute otolith-targeted vestibular stimulation is associated with condition-dependent metabolic differences across vestibulo–thalamo–striatal and sensorimotor-related regions in an MPTP-induced parkinsonian non-human primate. These exploratory findings demonstrate central metabolic engagement during stimulation but do not establish the functional significance, compensatory nature, or generalizability of the observed patterns. Replication in additional animals and complementary functional analyses will be required to determine their biological and therapeutic significance.

## Data Availability

Data pertaining to this study can be shared upon request to the following author RM-H. Requests to access the datasets should be directed to rmanrique@unav.es.
